# Prognostic value of systemic immune inflammation index and geriatric nutrition risk index in early-onset colorectal cancer

**DOI:** 10.3389/fnut.2023.1134300

**Published:** 2023-04-18

**Authors:** Shuai Xiang, Yu-Xiao Yang, Wen-Jun Pan, Ying Li, Jun-Hao Zhang, Yuan Gao, Shanglong Liu

**Affiliations:** ^1^Department of Gastrointestinal Surgery, Affiliated Hospital of Qingdao University, Qingdao, China; ^2^Department of Gastroenterology, China-Japan Friendship Hospital of Peking University, Beijing, China; ^3^Department of Blood Transfusion, Affiliated Hospital of Qingdao University, Qingdao, China

**Keywords:** systemic inflammation, early-onset colorectal cancer, overall survival, SII, GNRI

## Abstract

**Background:**

Systemic nutritional and inflammatory markers, which are easy to measure are associated with the progression and prognosis of many cancers. Nevertheless, among the various available indicators, optimal prognostic indicators for patients with early-onset colorectal cancer have not been identified. Therefore, the aim of this study was to identify optimal nutritional and inflammatory markers for early-onset colorectal cancer and examine the relationship between systemic nutritional and inflammatory markers before treatment and survival in patients with early-onset colorectal cancer.

**Methods:**

We retrospectively collected data from 236 eligible patients with early-onset colorectal cancer. Area under the prognostic curve (AUC) and concordance index (C-index) were used to compare seven systemic nutritional and inflammatory markers to identify the optimal inflammatory immune markers. Univariate and multivariate COX regression analyses were used to evaluate the prognostic value of indicators in the total study population and different subgroups.

**Results:**

The AUC and C-index showed that the systemic immune inflammation index (SII) and geriatric nutrition risk index (GNRI) had higher prognostic values than other systemic nutritional and inflammatory indicators. Compared with patients in the low SII group, those in the high SII group had lower overall survival (HR, 4.42, 95% CI, 2.36–8.27, *p* = 0.000). Compared with patients in the high GNRI group, those in the low GNRI group had lower overall survival (HR, 0.33, 95% CI, 0.19–0.56, *p* = 0.000). SII was negatively associated with GNRI (R = −0.3, *p* < 0.001), and both were correlated with the tumor stage.

**Conclusion:**

SII and GNRI are suitable nutritional and inflammatory factors for predicting OS in patients with early-onset colorectal cancer; high SII and low GNRI were correlated with worse prognoses. Identifying the high inflammatory state and low nutritional state of patients before surgery and conducting active and timely therapeutic interventions could improve patient prognosis.

## 1. Introduction

Colorectal cancer (CRC) is the fourth most deadly cancer globally, with almost 900,000 annual deaths (1). Due to the popularization of CRC screening in people over 50 years of age and lifestyle improvements, the overall incidence of and mortality from CRC have decreased by more than 45% since 1980 (2, 3). However, the incidence and mortality of colorectal cancer are increasing in adults aged 50 and younger (4, 5). Colorectal cancer diagnosed in people younger than 50 is generally considered early-onset, as screening programs begin at age 50 in most countries (6). Compared with late-onset colorectal cancer (older than 50 years), early-onset colorectal cancer presents with later stage tumors and unfavorable clinicopathological features; survival data on this group are currently lacking and contradictory (6). Analysis of the SEER database showed that younger patients are more prone to poorly differentiated, mucinous, and signet ring tumors than elderly patients (7). Although younger patients are more likely to receive neoadjuvant chemoradiotherapy and adjuvant chemotherapy, their disease-specific outcomes are comparable to those of older patients. This may be related to the unique biological and molecular characteristics of early-onset colorectal cancers (6, 8).

Increasing evidence has shown that inflammation is closely associated with cancer (9). McAllister and Weinberg (10) considered tumor-related systemic inflammation as the seventh feature of cancer, and only the “tip of the iceberg” in terms of cancer biology and treatment. All colorectal tumors that have been studied so far have been associated with the inflammatory environment. The inflammatory response plays a role in the entire process of tumorigenesis and cancer development. Inflammation induced by sporadic tumors can promote local tumor growth and distant metastases (9), which is generally reflected in increased levels of inflammatory cells and proinflammatory mediators. At the same time, pro-inflammatory cytokines produced by tumors will destroy the metabolism of carbohydrates, fats and proteins in the whole body, aggravate catabolism and lead to muscle decomposition. Combined with tumor consumption and insufficient nutrition intake, cancer patients have a high risk of malnutrition. Malnutrition can not only reduce the tolerance of cancer patients to anti-cancer treatment, including increasing the toxicity of treatment and impairing the quality of life, but also is closely related to the prognosis (11, 12). However, a recent European study found that only 30%–60% of cancer patients at risk of malnutrition received nutritional support treatment, meaning that many malnourished patients did not receive necessary nutritional interventions (13, 14). Hence, the search for nutritional and inflammatory biomarkers associated with poor prognosis is clinically important.

Systemic nutritional and inflammation response indicators are obtained by measuring clinical biochemical and hematological indicators. A variety of nutritional and inflammatory indicators including neutrophil-lymphocyte ratio (NLR) (15), platelet-lymphocyte ratio (PLR) (16), advanced lung cancer inflammation index (ALI) (17), systemic immune-inflammation index (SII) (18), geriatric nutrition risk index (GNRI) (19), prognostic nutritional index (20), and albumin to globulin ratio (AGR) (21) have been shown to be related to the prognosis of cancer. However, the prognostic role of these nutritional and inflammatory markers in early-onset CRC remains unclear.

Therefore, this study investigated optimal nutritional and inflammation indicators for early-onset colorectal cancer and examined the relationship between pre-treatment systemic nutritional and inflammatory indicators and survival rate. These factors are closely related to prognosis and could contribute to the risk stratification of patients.

## 2. Methods

### 2.1. Study population

We retrospectively collected data from patients younger than 50 years old at diagnosis who underwent radical resection of colorectal cancer in our hospital from December 2013 to December 2017. The inclusion criteria were as follows: (1) age at diagnosis between 18 and 49 years; (2) postoperative pathological diagnosis of adenocarcinoma; (3) had test indices before surgery or within 1 week before chemoradiotherapy. The exclusion criteria were as follows: (1) non-colorectal primary malignancy; (2) missing clinical data; (3) distant metastasis at the time of diagnosis. This study was approved by the Ethics Committee of our hospital. Informed consent was waived owing to the retrospective nature of the study.

### 2.2. Markers of systemic nutrition and inflammation

A variety of systemic nutritional and inflammatory markers that reportedly have prognostic value (all indicators were obtained within 1 week before surgery or other treatment) were retrospectively collected and calculated. The calculation formula was as follows. Inflammatory markers: NLR, neutrophil/lymphocyte (17); PLR, platelet/lymphocyte (16); ALI, BMI*albumin/NLR (17); SII, platelet*neutrophil/lymphocyte (18). Nutritional indices: GNRI, 1.489*albumin + 41.7*present body weight (PBW)/ideal body weight(IBW) (19); AGR, albumin/globulin (21); PNI, albumin+0.005*lymphocyte (20). BMI was defined as weight per height in meters squared. The IBW was defined as: for men = height − 100 − [(height − 150)/4]; for women = height − 100 − [(height − 150)/2.5].

### 2.3. Other covariates and end points

We also collected demographic information (age, gender, BMI, smoking history, drinking history), oncology information (tumor stage, tumor location, differentiation degree, nerve invasion status, vascular tumor thrombus), and treatment information (preoperative and postoperative radiotherapy and chemotherapy). Overall survival (OS) was the main study endpoint and was defined as the time between the initial diagnosis and death from any cause (the last follow-up was used for patients lost to follow-up; patients who were still alive at the end of the study were considered at the end of follow-up).

## 3. Statistics

SPSS 25.0 and R software (version 4.1.2) were used to analyze the data. Shapiro–Wilk test was used to test the normality of the distribution of continuous variables. Continuous variables were described as mean plus standard deviation (SD) or median (Q1 to Q3), depending on their distribution. For normally distributed data, the difference between the two groups was evaluated using Student’s t test, and the Mann–Whitney *U*-test was used otherwise. Categorical variables are presented as absolute numbers and percentages, and Fisher’s exact test and Pearson’s Chi-square test were used for comparisons between groups. The optimal cut-off value was calculated based on the maximally selected rank statistic in the “survminer” R package, which can determine the optimal cut-off value for one or multiple continuous variables at once. This is an outcome-oriented methods providing a value of a cut-off value that correspond to the most significant relation with outcome (here, overall survival). The best cut-off values of SII and GNRI were 637.6 and 83.13, respectively (Supplementary Figures S1, S2). The survival curve was drawn using the Kaplan–Meier method, and survival differences were compared using the Log-Rank test. Variables known to affect overall survival were included in the multivariate Cox proportional hazards model, and hazard ratios (HRs) and 95% confidence intervals were calculated. Three adjusted models were built: Model 0: unadjusted; Model 1: Adjusted for age, sex, BMI, and TNM stage; Model 2: Based on Model 1 and further adjusted for smoking status, alcohol consumption, tumor location, differentiation degree, nerve invasion status, vascular tumor thrombus, preoperative treatment, and postoperative treatment. An interaction *p* < 0.1 in the subgroup analysis was considered significant for the interaction. In other analyses, a two-sided *p* ≤ 0.05 was considered statistically different.

## 4. Results

### 4.1. Patient characteristics

A total of 236 eligible patients were recruited into the study (Supplementary Figure S3). The median patient age was 45 years; 72 patients (30.5%) were younger than 40 and 164 patients (69.5%) were 40–49 years old. In this study, 1-, 3-, and 5-year survival rates were 91.3%, 76.5%, and 65.7%, respectively. All the patients included were Han nationality. The baseline patient characteristics are summarized in Table 1.

**Table 1:** Baseline characteristics of the study population.

Characteristics	Overall patients	High SII	Low SII	*P-*value	High GNRI	Low GNRI	*P*-value
	(*n* = 236)	(≥637.6)	(<637.6)		(≥83.13)	(<83.13)	
		(*n* = 132)	(*n* = 104)		(*n* = 203)	(*n* = 33)	
Age, M (Q1~Q3), y	45 (39–48)	45 (39.3–48)	45 (38.25–47)	0.648	45 (39–47)	45 (38.5–48.5)	0.609
Gender, *n* (%)				0.000^*^			0.002^*^
Male	143 (60.6)	66 (50.0%)	77 (74.0%)		131 (64.5%)	12 (36.4%)	
Female	93 (39.4)	66 (50.0%)	27 (26.0%)		72 (35.5%)	21 (63.6%)	
BMI, M (Q1~Q3), kg/m^2^	22.9	22.3	23.3	0.051	22.8	24	0.119
	(20.8–25.6)	(20.5–25.0)	(21.2–26.3)		(20.8–25.1)	(20.8–27.7)	
BMI, *n* (%)				0.075			0.123
<18.5	15 (6.4)	10 (7.6%)	5 (4.8%)		13 (6.4%)	2 (6.1%)	
18.5–24	139 (58.9)	83 (62.9%)	56 (53.8%)		124 (61.1%)	15 (45.5%)	
24–28	58 (24.6)	24 (18.2%)	34 (32.7%)		49 (24.1%)	9 (27.3%)	
>28	24 (10.2)	15 (11.4%)	9 (8.7%)		17 (8.4%)	7 (21.2%)	
Smoking, *n* (%)				0.028^*^			0.176
No	184 (78.0)	110 (83.3%)	74 (71.2%)		155 (76.4%)	29 (87.9%)	
Yes	52 (22.0)	22 (16.7%)	30 (28.8%)		48 (23.6%)	4 (12.2%)	
Alcohol, *n* (%)				0.013^*^			0.344
No	190 (80.5)	114 (86.4%)	76 (73.1%)		161 (79.3%)	29 (87.9%)	
Yes	46 (19.5)	18 (13.6%)	28 (26.9%)		42 (20.7%)	4 (12.1%)	
Tumor stage, *n* (%)				0.017^*^			0.000^*^
I	32 (13.6)	13 (9.8%)	19 (18.3%)		32 (15.8%)	0 (0%)	
II	97 (41.1)	49 (37.1%)	48 (46.2%)		89 (43.8%)	8 (24.2%)	
III	107 (45.3)	70 (53.0%)	37 (35.6%)		82 (40.4%)	25 (75.8%)	
Tumor location, *n* (%)				0.761			0.155
Colon	141 (59.7)	80 (60.6%)	61 (58.7%)		125 (61.6%)	16 (48.5%)	
Rectum	95 (40.3)	52 (39.4%)	43 (41.3%)		78 (38.4%)	17 (51.5%)	
Differentiated degree, *n* (%)				0.359			0.032^*^
Poorly	75 (31.8)	47 (35.6%)	28 (26.9%)		59 (29.1%)	16 (48.5%)	
Moderately	153 (64.8)	81 (61.4%)	72 (69.2%)		138 (68.0%)	15 (45.5%)	
Well	8 (3.4)	4 (3.0%)	4 (3.8%)		6 (3.0%)	2 (6.1%)	
Preoperative therapy, *n* (%)				0.061			0.000^*^
No	184 (78.0)	97 (73.5%)	87 (83.7%)		167 (82.3%)	17 (51.5%)	
Yes	52 (22.0)	35 (26.5%)	17 (16.3%)		36 (17.7%)	16 (48.5%)	
Postoperative therapy, *n* (%)				0.174			0.002^*^
No	41 (17.4)	19 (14.4%)	22 (21.2%)		41 (20.2%)	0 (0%)	
Yes	195 (82.6)	113 (85.6%)	82 (78.8%)		162 (79.8%)	33 (100%)	
Nerve invasion, *n* (%)				0.005^*^			0.165
Negative	156 (66.1)	77 (58.3%)	79 (76.0%)		138 (68.0%)	18 (54.5%)	
Positive	80 (33.9)	55 (41.7%)	25 (24.0%)		65 (32.0%)	15 (45.5%)	
Intravascular tumor emboli, *n* (%)				0.98			0.296
Negative	170 (72.0)	95 (72.0%)	75 (72.1%)		149 (73.4%)	21 (63.6%)	
Positive	66 (28.0)	37 (28.0%)	29 (27.9%)		54 (26.6%)	12 (36.4%)	
NLR, M (Q1~Q3)	2.49 (1.73–3.47)	2.88 (2.24–3.87)	1.94 (1.45–2.67)	0.000^*^	2.35 (1.69–3.31)	2.81 (2.39–4.23)	0.017^*^
PLR, M (Q1~Q3)	173.9	198.9	145.3	0.000^*^	169.1	247.6	0.004^*^
	(130.1–233.4)	(156.3–247.9)	(121.9–194.4)		(126.9–225.8)	(148.2–278.9)	
ALI, M (Q1~Q3)	44.3 (29.1–65.5)	36.0 (23.4–52.5)	59.7 (43.8–83.5)	0.000^*^	44.9 (29.1–67.1)	36.4 (24.9–57.5)	0.098
SII, M (Q1~Q3)	691.2 (437.1–891.1)	/	/	/	674.1 (405.7–862.0)	846.8 (594.5–1103.9)	0.010^*^
GNRI, M (Q1~Q3)	100.4 (91.4–107.7)	96.1 (87.3–104.5)	104.9 (96.5–110.6)	0.000^*^	/	/	/
AGR, M (Q1~Q3)	1.51 (1.28–1.71)	1.42 (1.21–1.66)	1.57 (1.38–1.75)	0.002^*^	1.51 (1.29–1.71)	1.42 (1.20–1.68)	0.449
PNI, M (Q1~Q3)	49.4 (43.5–55.4)	48.6 (43.4–56.8)	50.3 (44.3–54.9)	0.842	49.4 (43.5–55.2)	48.0 (43.6–62.8)	0.858

M (Q1~Q3), median (Q1~Q3); BMI, body mass index; NLR, neutrophil to lymphocyte ratio; PLR, platelet to lymphocyte ratio; ALI, advanced lung cancer inflammation index; SII, systemic immune inflammation index; GNRI, geriatric nutrition risk index; AGR, albumin to globulin ratio; PNI, prognostic nutritional index. *p ≤ 0.05.

### 4.2. Selection of the best prognostic nutritional and inflammatory index

**Figure 1:**
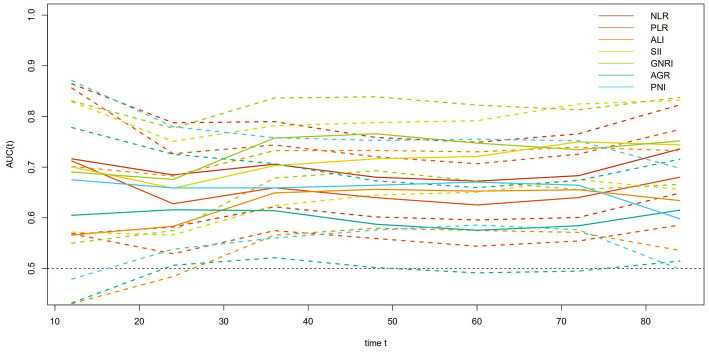
Time-dependent ROC for the seven immune-inflammatory markers.

The optimal prognostic nutritional and inflammatory index in patients with early-onset colorectal cancer was selected through time-dependent ROC and concordance index (C-index). The results showed that SII and GNRI had higher prognostic values than other nutritional and inflammatory indicators; C-index and 95% CI were 0.692 (0.633–0.750) and 0.711 (0.652–0.770), respectively (Figure 1; Supplementary Table S1). Based on the SII cutoff value, all patients were divided into High SII and Low SII groups. The baseline characteristics are shown in Table 1. There were significant differences in gender, smoking status, alcohol consumption, tumor stage, and neurological invasion status, NLR, PLR, ALI, GNRI and AGR between the two groups (all *p* ≤ 0.05). All patients were divided into High GNRI and Low GNRI groups based on the GNRI cut-off value. The baseline characteristics are shown in Table 1. Two groups had significant differences in gender, tumor stage, tumor differentiation, preoperative adjuvant therapy, postoperative adjuvant therapy, NLR, PLR and SII (all *p* ≤ 0.05). We also observed a significant negative correlation between SII and GNRI (R = −0.3, *p* < 0.001; Supplementary Figure S4).

### 4.3. Prognostic value of SII and GNRI in early-onset colorectal cancer

**Figure 2:**
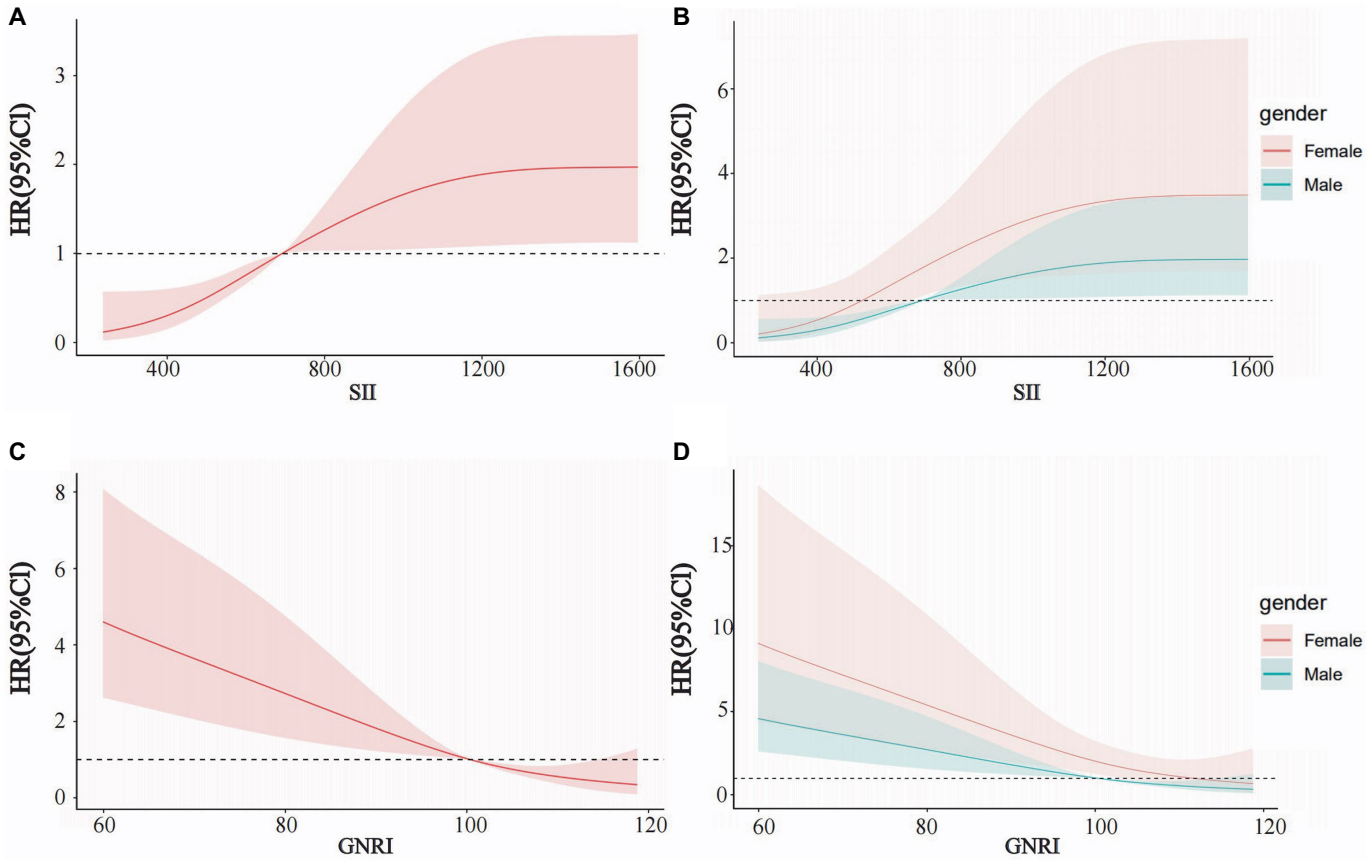
Restricted cubic spline curves for SII and GNRI in EOCRC. **(A)** SII in all patients, **(B)** SII in males and females, **(C)** GNRI in all patients, and **(D)** GNRI in males and females.

**Figure 3:**
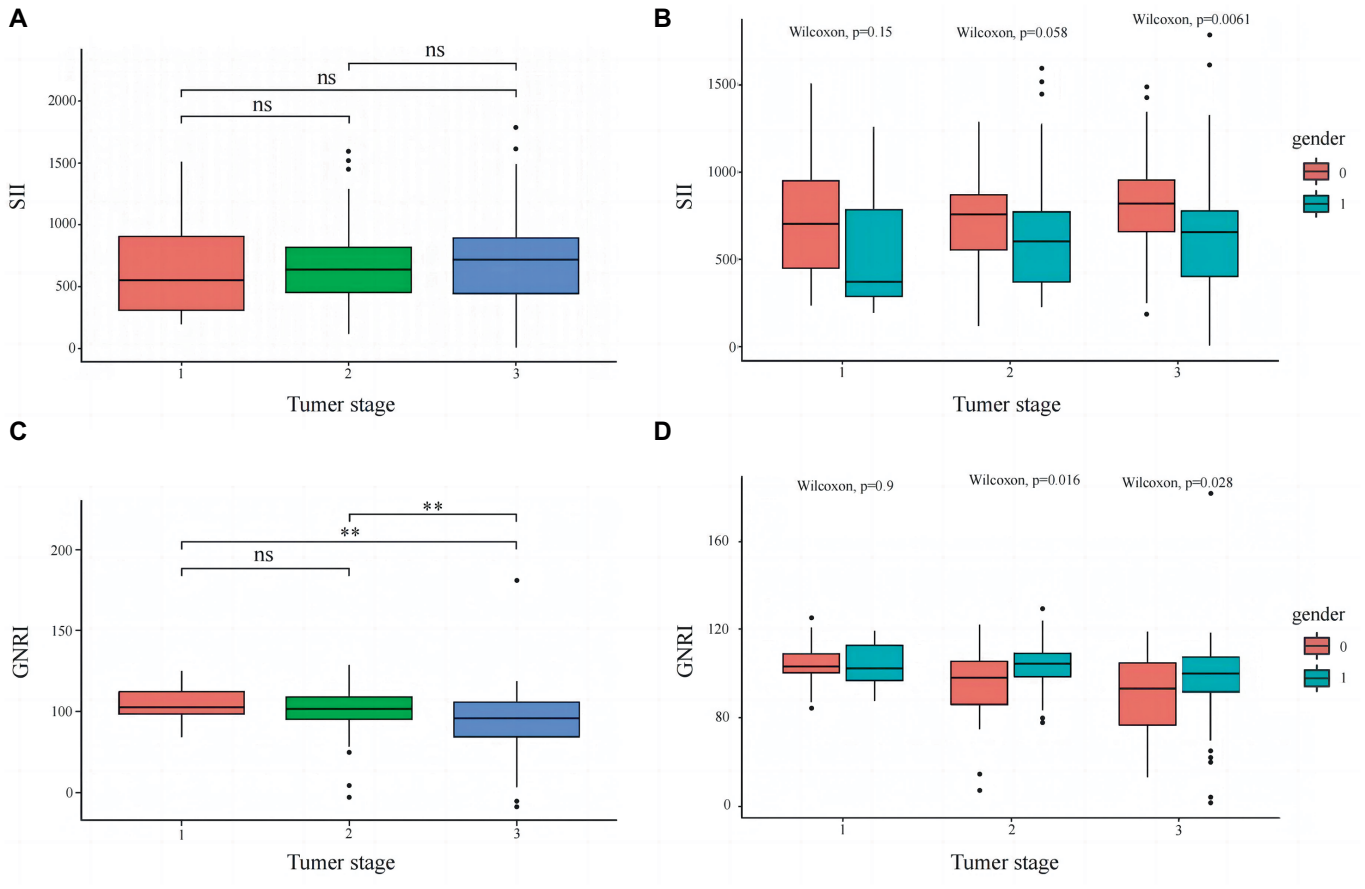
Distribution of SII and GNRI in different tumor stages. **(A)** SII in all patients, **(B)** SII in males and females, **(C)** GNRI in all patients, and **(D)** GNRI in males and females. **p ≤ 0.05.

**Figure 4:**
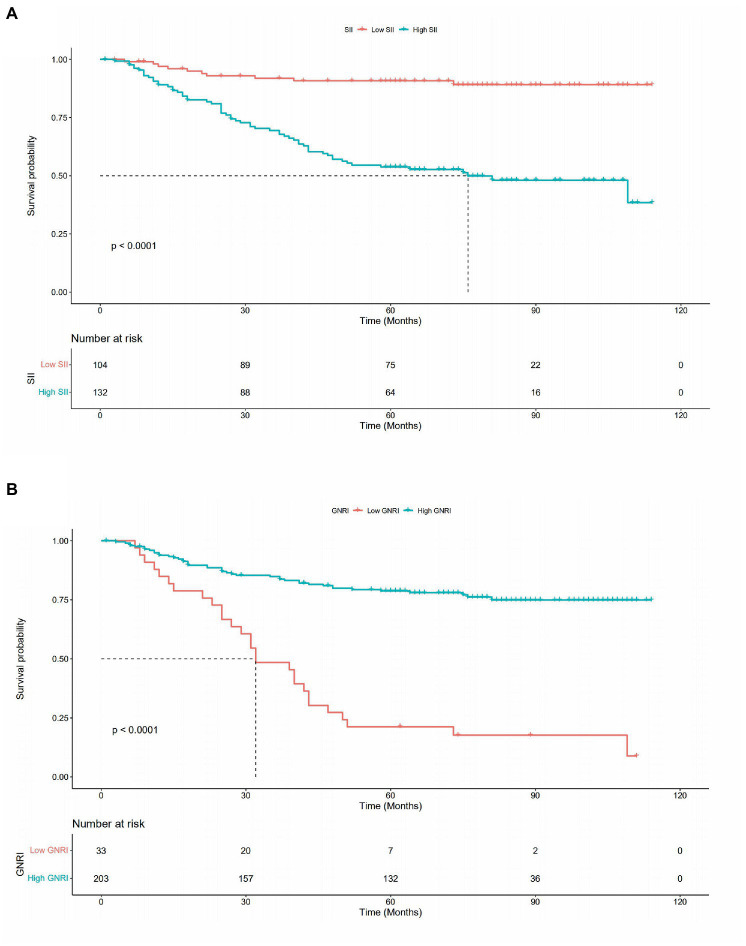
Kaplan–Meier survival curve for SII and GNRI in EOCRC. **(A)** SII **(B)** GNRI.

Restricted cubic spline (RCS) was used to evaluate the relationship between SII, GNRI, and patient HR. The results indicated that with an increase in SII and a decrease in GNRI, patient HR gradually increased, suggesting that the risk of death gradually increased (Figures 2A,C). Consistent results were observed in the gender subgroups (Figures 2B,D). The box plot shows that as SII gradually increased, the tumor stage also increased (Figure 3A); GNRI gradually decreased with increasing tumor stage. There were statistical differences between stages 1 and 3, and stages 2 and 3 (Figure 3C). Consistent results were observed in the gender subgroups (Figures 3B,D), which may partially explain the relationship between SII, GNRI, and HR. The survival curve showed that compared to patients with low SII, those with high SII had a worse prognosis (Figure 4A, *P* < 0.0001). For every SD increase in SII, the risk of death increased 1.08-fold (Table 2, model 2, 95% CI = 1.05–1.11, *p* = 0.000). Compared to patients with low SII, the risk of death in patients with high SII increased 4.42-fold (model 2, 95% CI = 2.36–8.27, *p* = 0.000). Patients were divided into four groups (Q1: ~437.93; Q2: 437.93–691.19; Q3: 691.19–890.71; Q4: 890.71) according to the SII quartile value. The multivariate COX regression model showed that patients in the Q2 (model 2, HR = 4.09, 95% CI = 1.47–11.37, *p* = 0.006), Q3 (model 2, HR = 3.97, 95% CI = 1.45–10.86, *p* = 0.007) and Q4 (model 2, HR = 8.49, 95% CI = 3.22–22.36, *p* = 0.000) groups had an increased risk of death compared to those in the Q1 group. Sensitivity analysis results showed similar results, excluding patients who died within a year (Supplementary Table S2). However, patients with high GNRI had a better prognosis compared to those with low GNRI (Figure 4B, *P* < 0.0001). For each standard deviation increase in GNRI, the risk of death was reduced 0.97-fold (Table 3, model 2, 95% CI = 0.96–0.98, p = 0.000). Compared to patients with low GNRI, the risk of death in those with high GNRI was decreased 0.33 times (model 2, 95% CI = 0.19–0.56, *p* = 0.000). Patients were divided into four groups (Q1: ~91.50; Q2: 91.50–100.37; Q3: 100.37–107.64; Q4: 107.64) according to the GNRI quartile value. The multivariate COX regression model showed that patients in Q3 (model 2, HR = 0.29, 95% CI = 0.14–0.59, *p* = 0.001) and Q4 (model 2, HR = 0.29, 95% CI = 0.13–0.64, *p* = 0.002) groups had a lower risk of death compared with those in the Q1 group. After the exclusion of patients who died within a year, the results of the sensitivity analysis suggested a similar survival outcome (Supplementary Table S3).

**Table 2:** Univariate and multivariate analysis on the OS of SII.

Variables	OS (model 0)^a^		OS (model 1)^b^		OS (model 2)^c^	
	Crude HR (95%CI)	Crude *P*	Adjusted HR (95%CI)	Adjusted *P*	Adjusted HR (95%CI)	Adjusted *P*
As continuous (per SD)	1.07 (1.04–1.09)	0.000^*^	1.05 (1.03–1.10)	0.000*	1.08 (1.05–1.11)	0.000*
**By SII cut-off**						
≤637.6	/	/	/	/	/	/
>637.6	5.27 (2.91–9.54)	0.000^*^	4.22 (2.29–7.77)	0.000^*^	4.42 (2.36–8.27)	0.000^*^
**By SII interquartile**						
Q1 (~437.93)	/	/	/	/	/	/
Q2 (437.93–691.19)	3.74 (1.38–10.16)	0.009^*^	3.57 (1.31–9.75)	0.012^*^	4.09 (1.47–11.37)	0.006^*^
Q3 (691.19–890.71)	5.46 (2.08–14.33)	0.001^*^	3.93 (1.47–10.48)	0.006^*^	3.97 (1.45–10.86)	0.007^*^
Q4 (890.71~)	9.43 (3.69–24.09)	0.000^*^	7.19 (2.79–18.55)	0.000^*^	8.49 (3.22–22.36)	0.000^*^

SII, systemic immune inflammation index; OS, overall survival; HR, hazards ratio; CI, confidence interval;^a^Model 0: Unadjusted.^b^Model 1: Adjusted for age, gender, BMI and tumor stage.^c^Model 2: Adjusted for age, gender, BMI, tumor stage, smoking, alcohol, tumor location, differentiated degree, nerve invasion, intravascular tumor emboli, preoperative therapy and postoperative therapy.

*p ≤ 0.05.

**Table 3:** Univariate and multivariate analysis on the OS of GNRI.

Variables	OS (model 0)		OS (model 1)		OS (model 2)	
	Crude HR (95%CI)	Crude *P*	Adjusted HR (95%CI)	Adjusted *P*	Adjusted HR (95%CI)	Adjusted *P*
As continuous (per SD)	0.96 (0.95–0.97)	0.000^*^	0.97 (0.96–0.98)	0.000*	0.97 (0.96–0.98)	0.000^*^
**By GNRI cut-off**						
≤83.1	/	/	/	/	/	/
>83.1	0.23 (0.14–0.36)	0.000^*^	0.35 (0.21–0.58)	0.000^*^	0.33 (0.19–0.56)	0.000^*^
**By GNRI interquartile**						
Q1 (~91.50)	/	/	/	/	/	/
Q2 (91.50–100.37)	0.67 (0.41–1.13)	0.134^*^	0.85 (0.50–1.47)	0.582	0.70 (0.39–1.23)	0.219
Q3 (100.37–107.64)	0.23 (0.12–0.46)	0.000^*^	0.30 (0.15–0.61)	0.001^*^	0.29 (0.14–0.59)	0.001^*^
Q4 (107.64~)	0.21 (0.10–0.44)	0.000^*^	0.28 (0.13–0.62)	0.002^*^	0.29 (0.13–0.64)	0.002^*^

SII, GNRI, geriatric nutrition risk index; OS, overall survival; HR, hazards ratio; CI, confidence interval;^a^Model 0: Unadjusted.^b^Model 1: Adjusted for age, gender, BMI and tumor stage.^c^Model 2: Adjusted for age, gender, BMI, tumor stage, smoking, alcohol, tumor location, differentiated degree, nerve invasion, intravascular tumor emboli, preoperative therapy and postoperative therapy.

*p ≤ 0.05.

**Figure 5:**
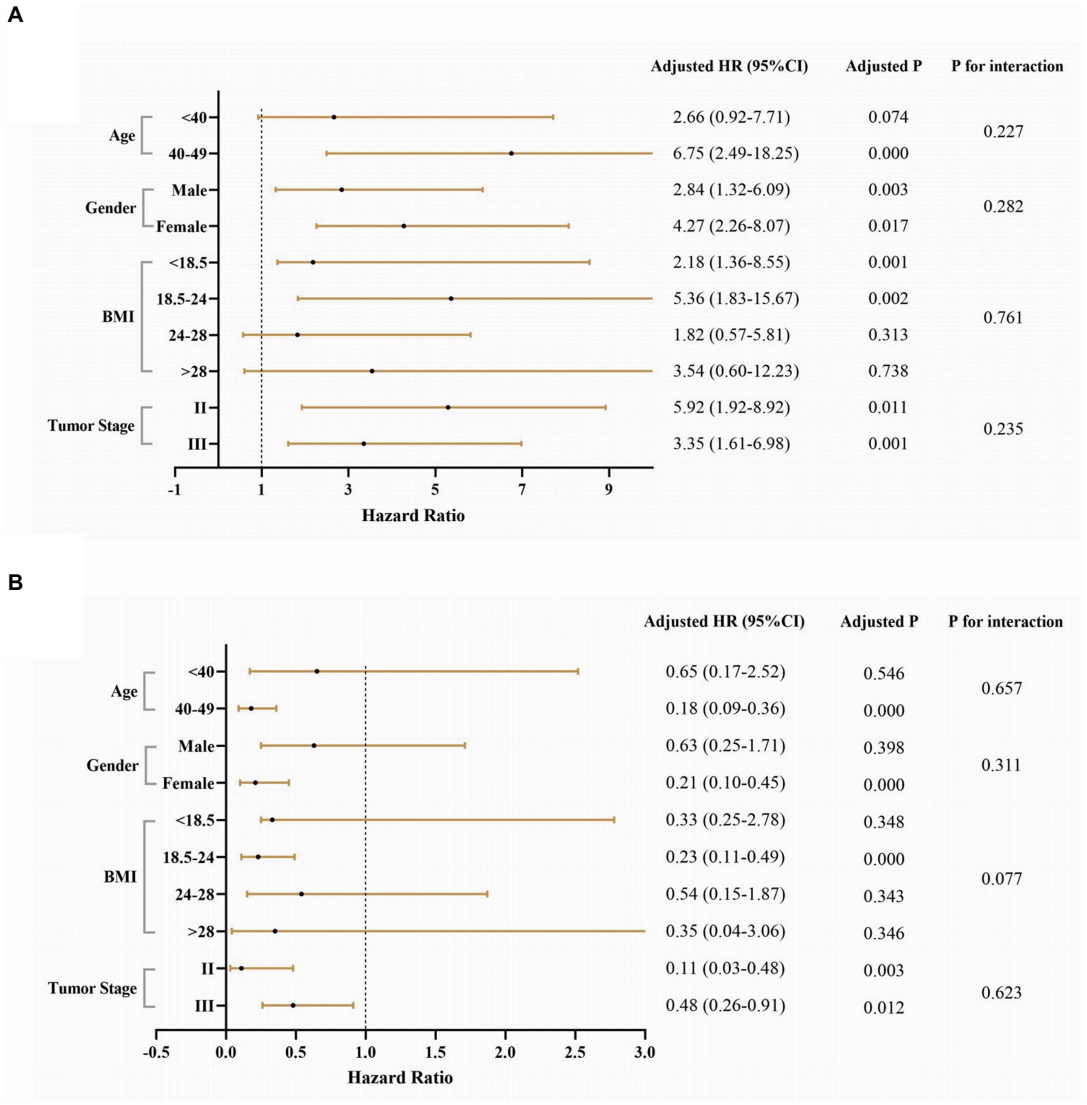
Stratification analysis of SII and GNRI in EOCRC. **(A)** High SII and low SII and **(B)** High GNRI and low GNRI. Adjusted for age, gender, BMI, tumor stage, smoking, alcohol, tumor location, differentiated degree, nerve invasion, intravascular tumor emboli, preoperative therapy, and postoperative therapy. Because the number of outcome events (deaths) in the stage I group was zero, we did not analyze this subgroup.

Subgroup analysis of SII showed significant prognostic value in patients except for those aged <40 years, BMI 24–28, and BMI > 28 (Figure 5A). We also observed that GNRI had a significant prognostic value in patients aged 40–49 years, female, with a BMI between 18.5–24, and tumor stages II and III (Figure 5B). Furthermore, SII and GNRI showed good survival prediction in the BMI subgroups (18.5–24, 24–28, >28), gender (male, female), vascular tumor thrombus (positive, negative), neural invasion (positive, negative), preoperative adjuvant therapy (yes, no), and postoperative adjuvant therapy (yes; Supplementary Figures S5, S6).

## 5. Discussion

Previous studies have shown that inflammatory mediators secreted due to chronic inflammation and related immune cells support the establishment and progression of tumors by inducing neoplastic mutations, increasing the proliferation rate of tumor cells, stimulating angiogenesis, and recruiting fibroblasts and other stromal cells (22–24). Some evidence is mounting that aspirin can reduce the incidence and growth rate of several cancers in animal models, mediated in part by the inhibition of COX-2 and a reduction in prostaglandins and other inflammatory mediators (25, 26). Notwithstanding various systemic inflammatory response (SIR) indicators reportedly related to cancer prognosis, optimal indicators in patients with early-onset colorectal cancer remain unclear. Our study found that SII and GNRI have potential prognostic value in patients with early-onset colorectal cancer.

Previous studies have shown that high SII is associated with poor prognosis in a variety of solid tumors (27–29). The formula for SII includes platelets, neutrophils, and lymphocytes. Increased SII generally reflects increased platelets and neutrophils or decreased lymphocytes, and its prognostic effect can be explained by the role each of these immune cells plays. Neutrophils recruited to inflammatory areas increase DNA damage and angiogenesis by producing large amounts of ROS, reactive nitrogen species (RNS), and MMP-9. Additionally, neutrophils suppress T cell viability through arginine depletion via arginase 1 (ARG1) and downregulation of CD3ζ (30). Moreover, neutrophils can also recruit macrophages and Tregs to promote tumor progression (31). Tumor necrosis factor (TNF) and cathepsin G derived from neutrophils promote distant metastasis of malignant tumors (32). Recently, studies have shown that platelets are not only the main cellular components of blood clots but also play an essential role in cancer growth and dissemination. Platelets are recruited to the tumor microenvironment to promote tumor-related blood coagulation, covering the tumor surface to protect tumor cells from the immune response. Related experiments have affirmed that specific blocking of platelet receptors such as GP1b/IX/V, GPIIbIIIa, and GPVI reduces the occurrence of metastasis (33). Lymphocytes, the most important immune cells in the body, play an anti-tumor role mainly by inducing lysis and apoptosis of target cells (34). During an inflammatory response, neutrophils suppress the immune system by inhibiting the cytolytic activity of lymphocytes, activated T cells, and natural killer cells. The lower the lymphocyte level, the worse the immune function. Isabelle et al. demonstrated that lymphopenia is an independent prognostic factor for overall and progression-free survival in a variety of cancers (35). Moreover, we found that with an increase in the tumor stage, the level of SII gradually increased; this trend was observed in both genders. NLR also showed relatively good predictive capacity in our study (AUC = 0.666). However, the predictive capacity of NLR was not as effective as that of SII. Compared with NLR, SII contains three types of inflammatory cells, more comprehensively reflecting the relationship between inflammation and immunity. Hence, an increase in SII indirectly reflects a decline in host immune function and increased tumor invasiveness (36).

GNRI is an indicator of nutritional status based on albumin, current body weight, and ideal body weight. It simulates changes in body weight through the ratio of current body weight to ideal body weight. GNRI was originally designed for elderly patients but is also suitable for young adults (37–39). Preoperative malnutrition is highly prevalent in patients with gastrointestinal (GI) cancer and can lead to increased postoperative complications, longer hospital length of stay (LOS), and worse prognosis (40, 41). Therefore, it is necessary to evaluate and improve the nutritional status of patients before treatment. Albumin is synthesized in the liver, and low albumin levels are often associated with malnutrition and tumor progression (42). Various cytokines such as IL-6 and TNF can increase catabolism and reduce albumin synthesis in cancer patients. In our study, GNRI gradually decreased with increasing tumor stage, which may have been related to poor nutritional status and tumor progression. In addition, we found a significant negative correlation between GNRI and SII (R = −0.3, *p* < 0.001). With the gradual increase in SII, GNRI gradually decreased. Proinflammatory cytokines and growth factors can promote host catabolism and lead to muscle breakdown as part of the anti-tumor systemic inflammatory response (43). Low muscle strength can also lead to local inflammation of the muscle, which further leads to muscle breakdown and aggravates the systemic inflammatory response (44). Shlomit et al. (45) noted that in patients with solid tumors, a lower skeletal muscle index (SMI) at the time of cancer diagnosis was associated with a poorer survival rate and could be used as a prognostic indicator. George et al. (46) indicated that compared to patients with normal albumin levels, patients with reduced albumin levels had a significantly lower skeletal muscle index and visceral fat index at the L3 level. Thus, we speculate that GNRI reflects the muscle level of patients to a certain extent.

Identification of a high inflammatory state and low nutritional status in patients before surgery are of great clinical significance. Therefore, positive and timely therapeutic intervention can improve prognosis. Endurance- and resistance-type exercises can maintain skeletal muscle mass and function as well as energy balance (46). Recent studies have shown that to counteract catabolic effects, n-3 fatty acids can be used to reduce muscle loss (47), non-selective anti-inflammatory drugs can be used to alleviate the inflammatory response (48), and protein intake should be increased (49).

Because this was a retrospective study, certain limitations should be taken into consideration. First, due to missing data, we could not examine other markers of systemic inflammation such as lymphocyte-C reactive protein ratio and C-reactive protein/albumin ratio. Second, the study population was patients with early-onset colorectal cancer, which limits the generalizability of the results to other age groups and other tumor types. Third, the possibility of residual and unmeasured confounding could not be completely ruled out because of the retrospective nature of the study. Finally, this was a single-center retrospective study with small sample size and unbalanced distribution between GNRI groups may have a potential impact on the results. Therefore, multi-center prospective studies are needed to confirm the effectiveness and prognostic ability of these nutritional and inflammatory markers.

## Data availability statement

The raw data supporting the conclusions of this article will be made available by the authors, without undue reservation.

## Ethics statement

The studies involving human participants were reviewed and approved by the Ethics Committee of the Affiliated Hospital of Qingdao University. Written informed consent for participation was not required for this study in accordance with the national legislation and the institutional requirements.

## Author contributions

SL and YG: conceptualization and supervision. SX, Y-XY, and W-JP: data curation, methodology, and software. SX and J-HZ: writing original draft. SL: review and editing. All authors contributed to the article and approved the submitted version.

## Funding

The study was supported by the National Natural Science Foundation of China (grant no. 81802888), the Key Technology Research and Development Program of Shandong (no. 2018GSF118088), the General Financial Grant from the China Postdoctoral Science Foundation (no. 2016M592143), and the Shandong Provincial Natural Science Foundation (no. ZR2022MH252).

## Conflict of interest

The authors declare that the research was conducted in the absence of any commercial or financial relationships that could be construed as a potential conflict of interest.

## Publisher’s note

All claims expressed in this article are solely those of the authors and do not necessarily represent those of their affiliated organizations, or those of the publisher, the editors and the reviewers. Any product that may be evaluated in this article, or claim that may be made by its manufacturer, is not guaranteed or endorsed by the publisher.

## Supplementary material

SUPPLEMENTARY FIGURE S1 The optimal cut-off value of SII based on the survminer R software package.

SUPPLEMENTARY FIGURE S2 The optimal cut-off value of GNRI based on the survminer R software package.

SUPPLEMENTARY FIGURE S3 Pathway for patient recruitment and selection.

SUPPLEMENTARY FIGURE S4 Significant negative correlation between SII and GNRI.

SUPPLEMENTARY FIGURE S5 Survival prediction of SII in different subgroups.

SUPPLEMENTARY FIGURE S6 Survival prediction of GNRI in different subgroups.
