# Supplementary figures and images for: Prognostic value of systemic immune inflammation index and geriatric nutrition risk index in early-onset colorectal cancer
Source: Front Nutr. 2023 Apr 18;10:1134300. doi: 10.3389/fnut.2023.1134300 (PMC10151795; doi:10.3389/fnut.2023.1134300)

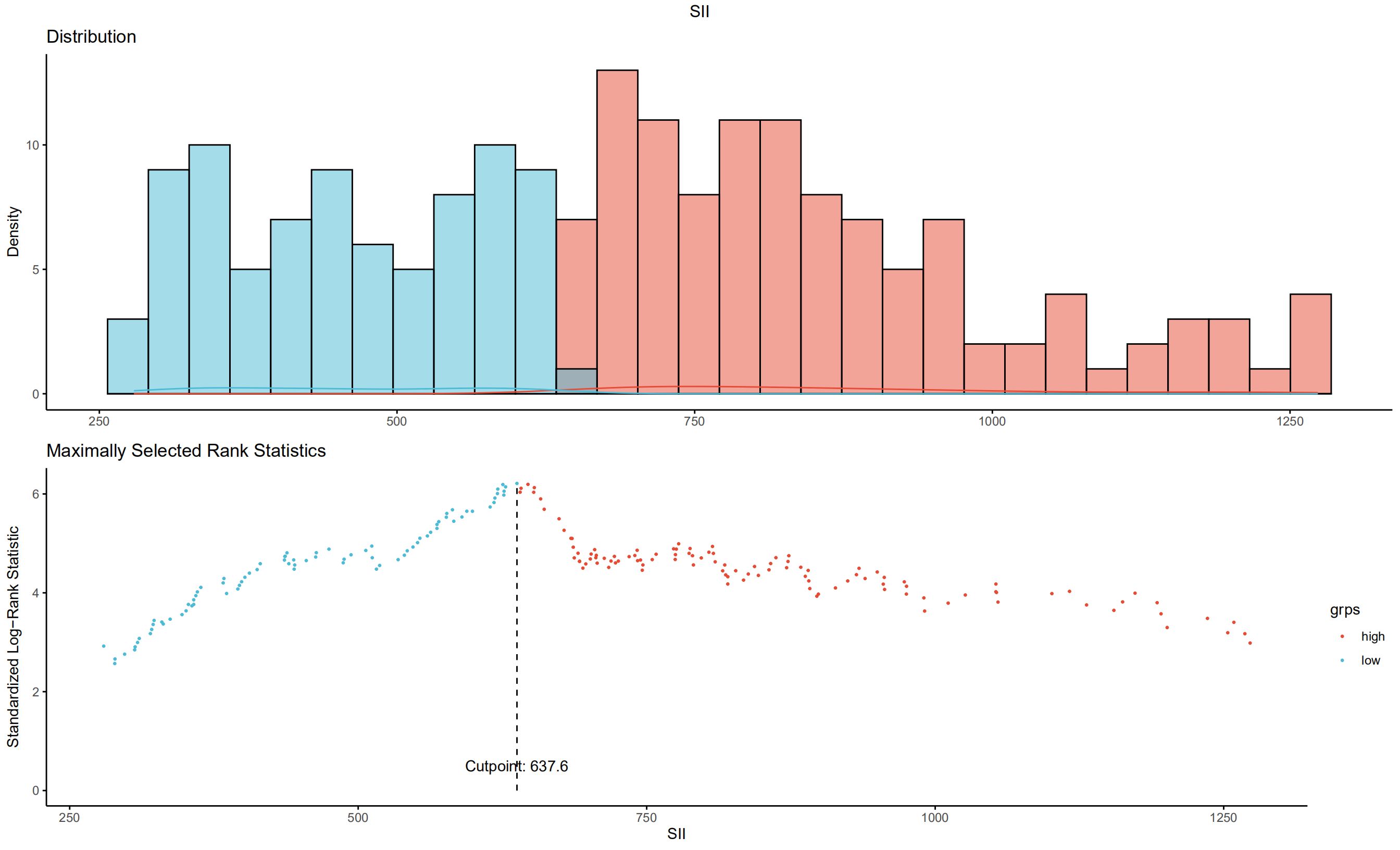

Supplement: SUPPLEMENTARY FIGURE S1 — The optimal cut-off value of SII based on the survminer R software package. [file Image_1.JPEG]

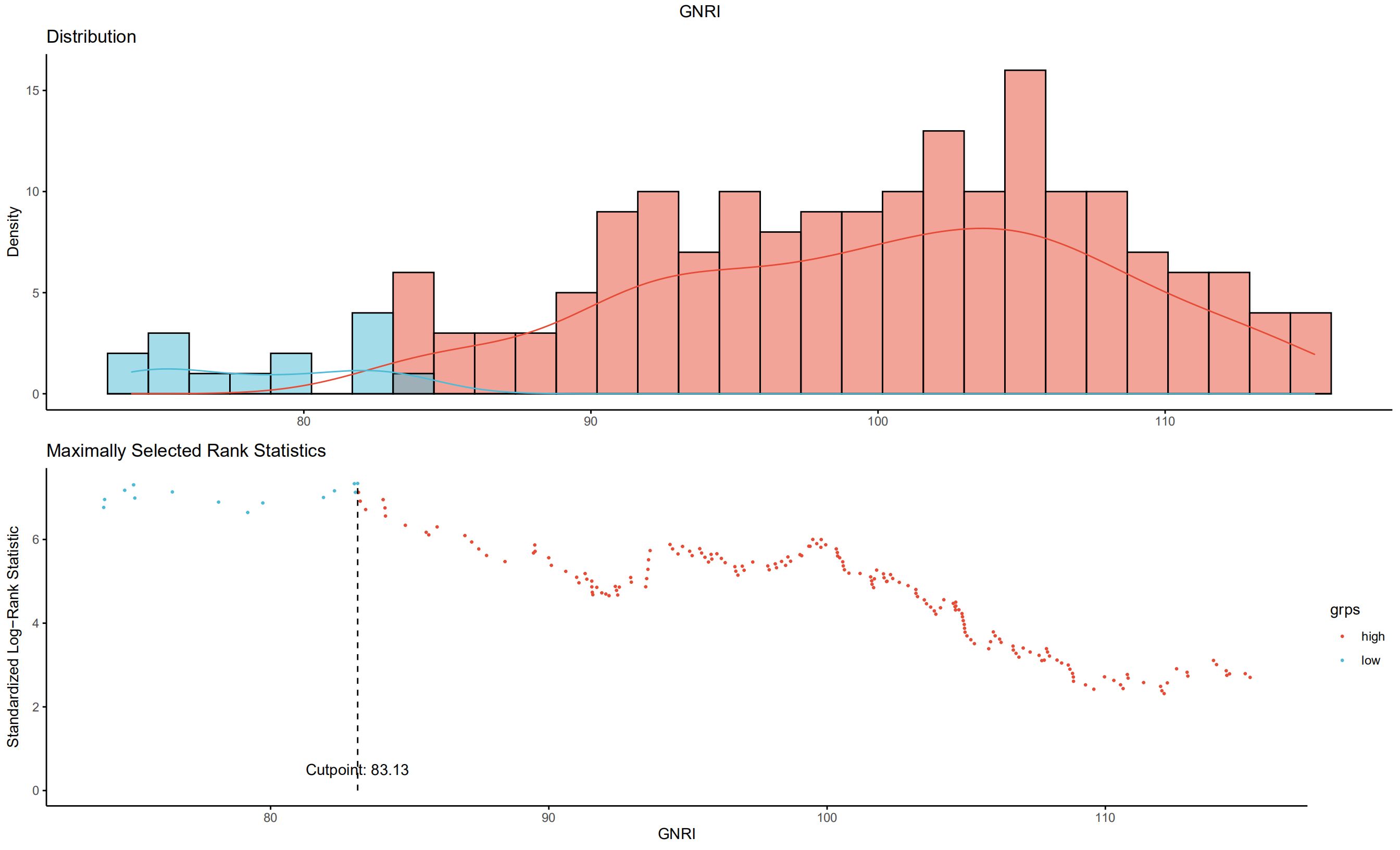

Supplement: SUPPLEMENTARY FIGURE S2 — The optimal cut-off value of GNRI based on the survminer R software package. [file Image_2.JPEG]

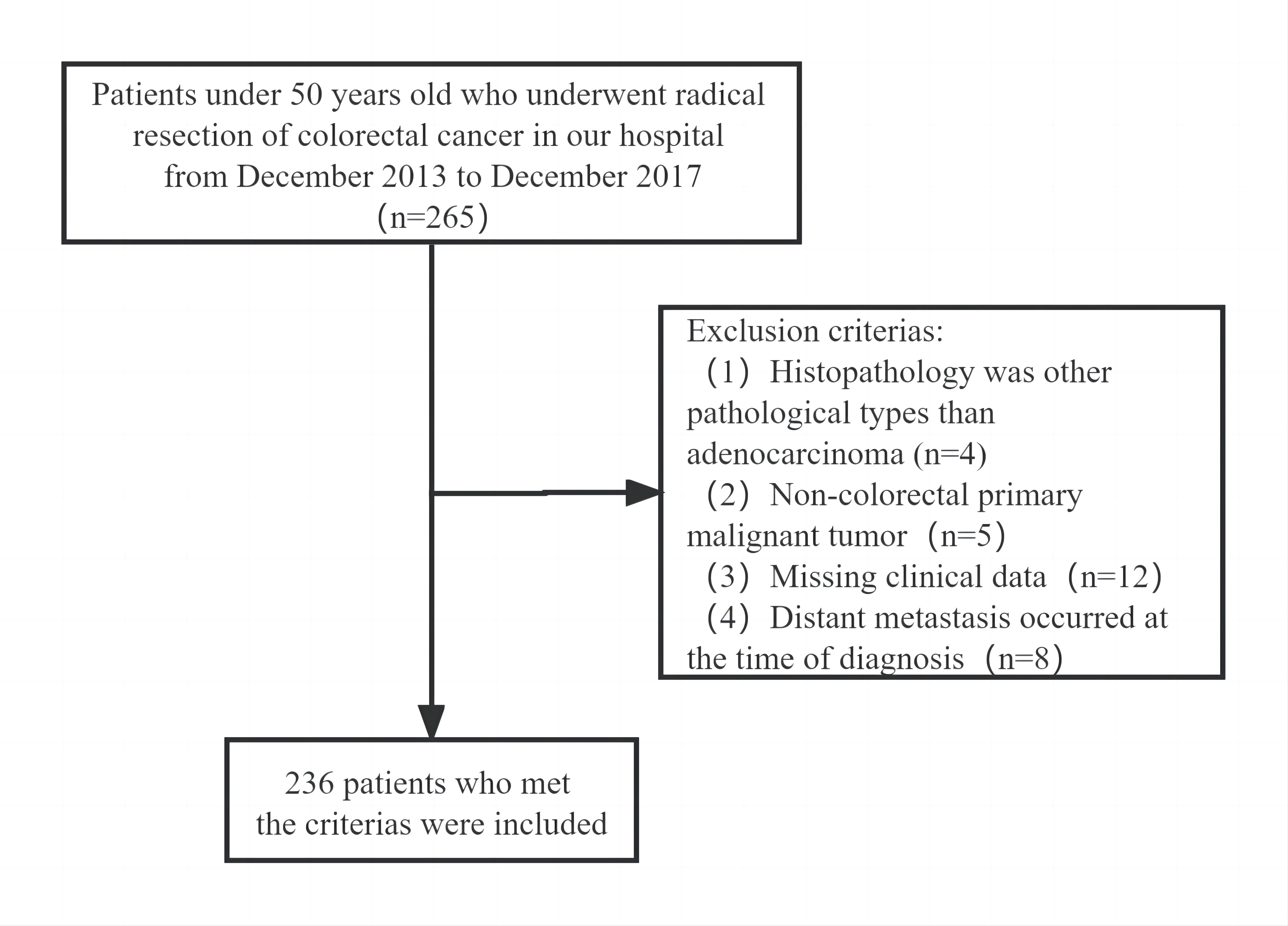

Supplement: SUPPLEMENTARY FIGURE S3 — Pathway for patient recruitment and selection. [file Image_3.JPEG]

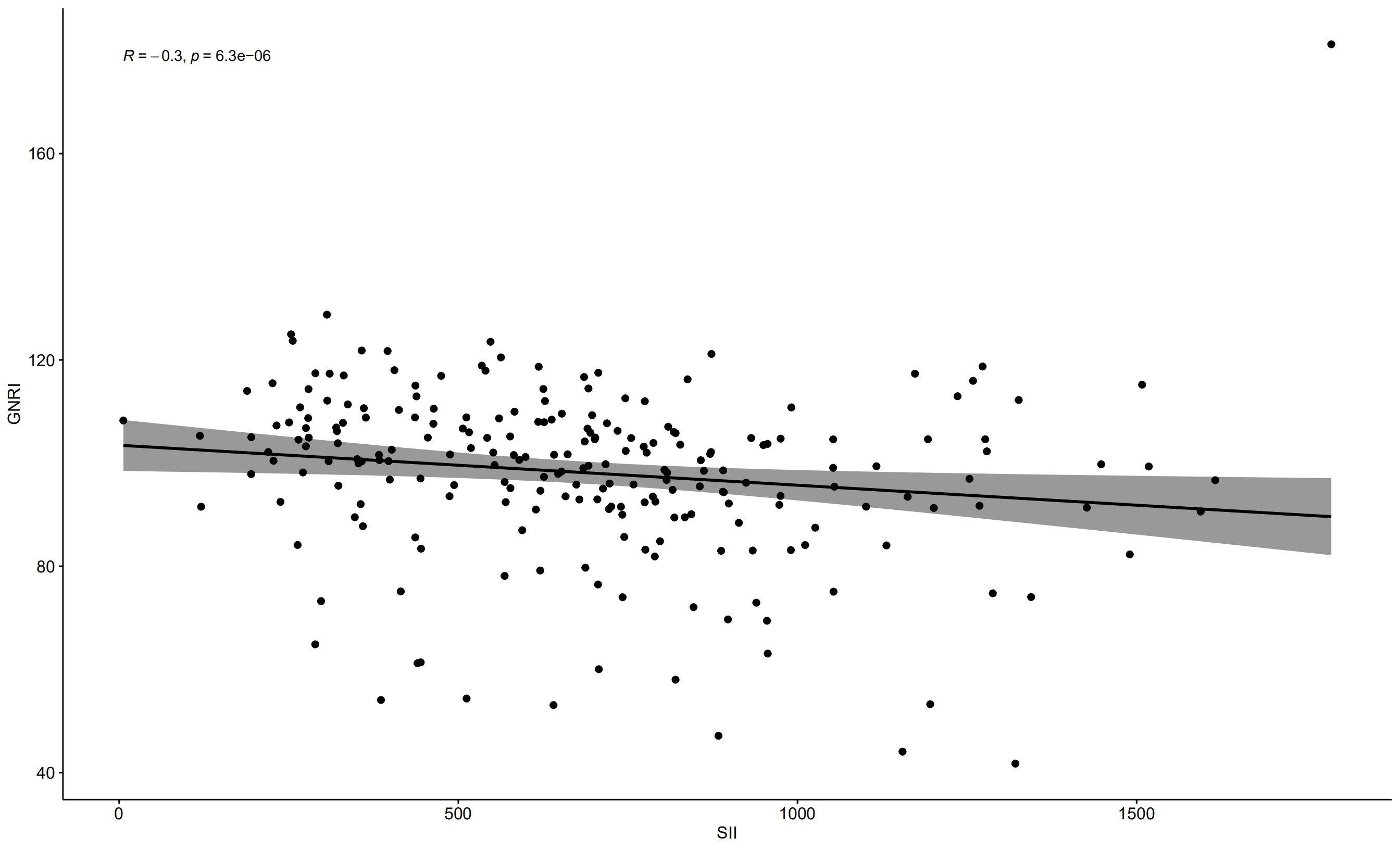

Supplement: SUPPLEMENTARY FIGURE S4 — Significant negative correlation between SII and GNRI. [file Image_4.JPEG]

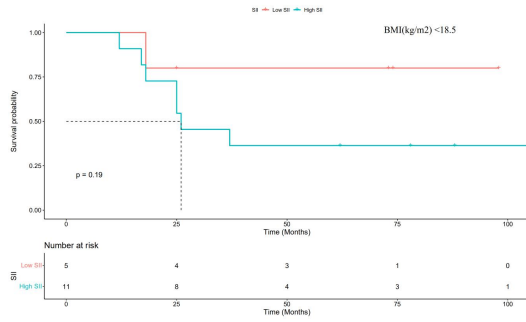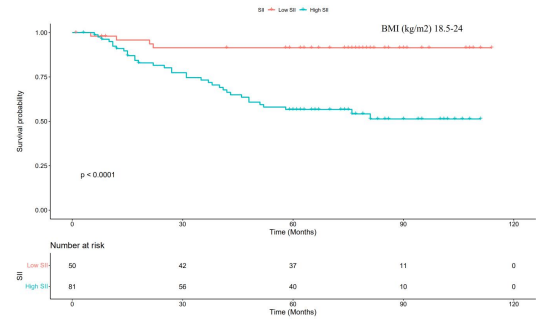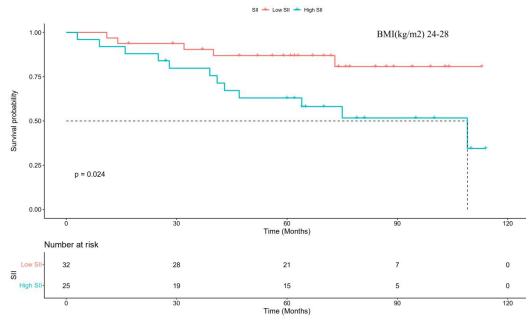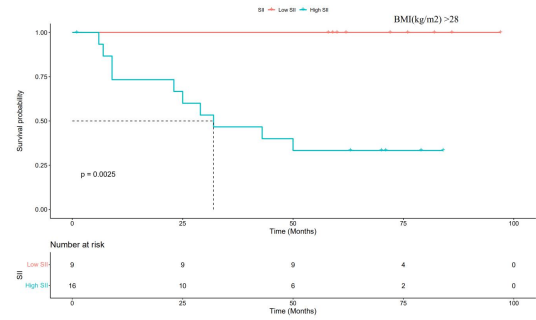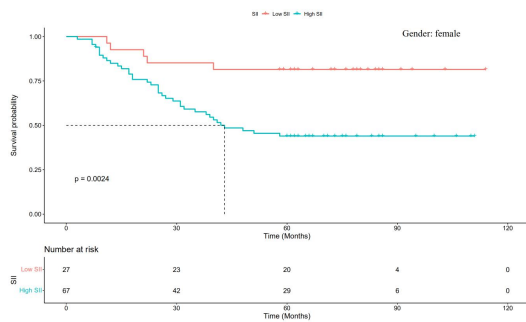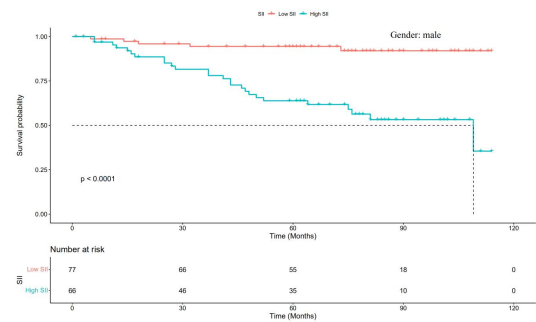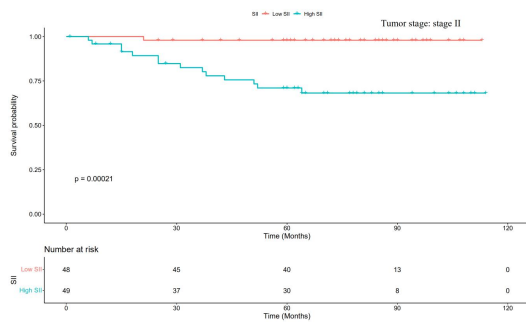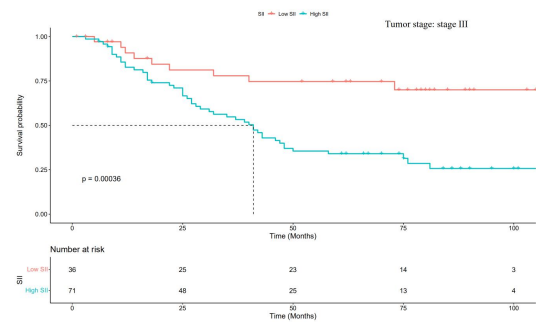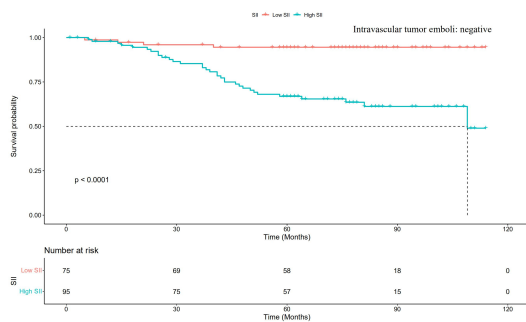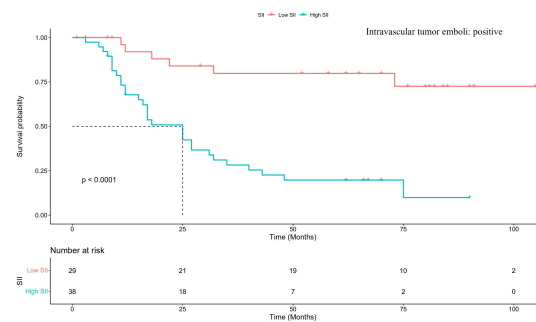

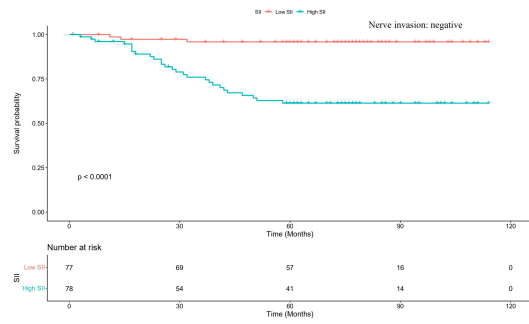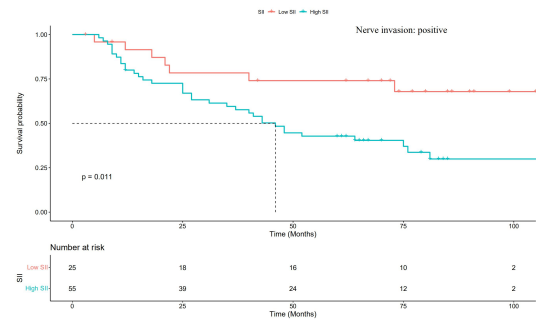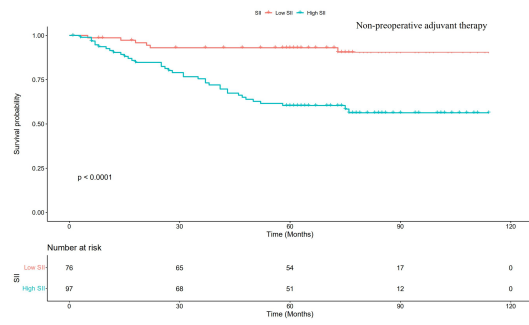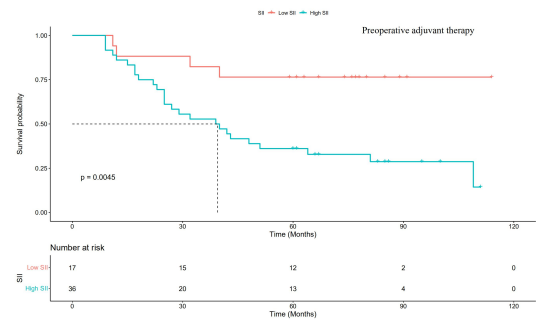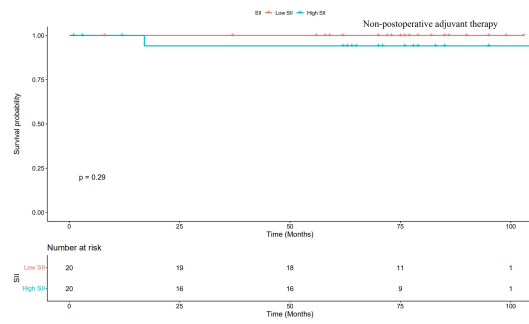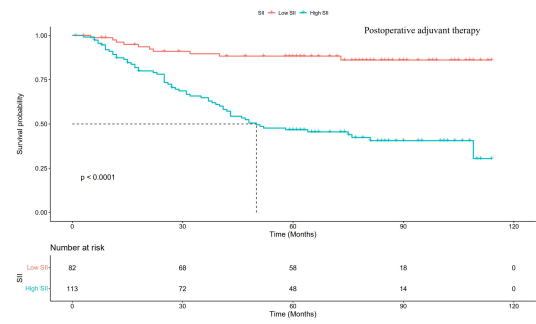

Supplement: SUPPLEMENTARY FIGURE S5 — Survival prediction of SII in different subgroups. [file Image_5.pdf]

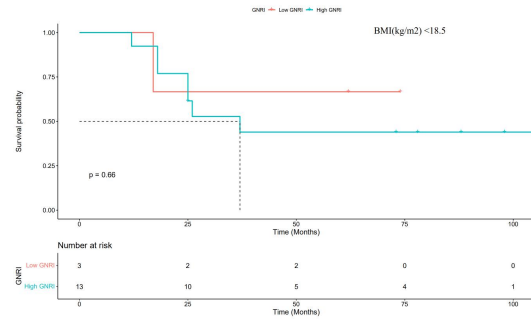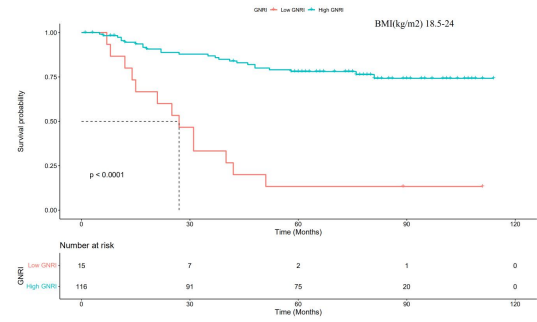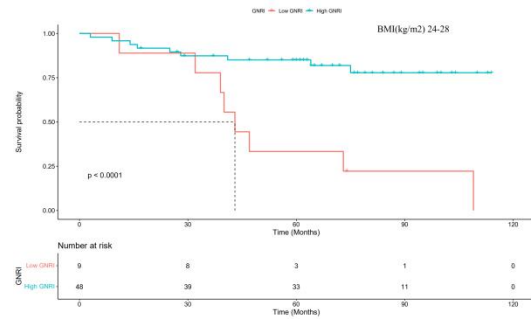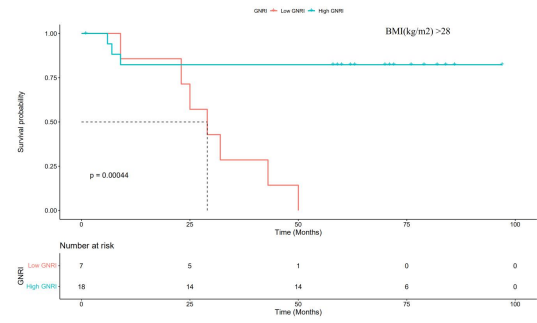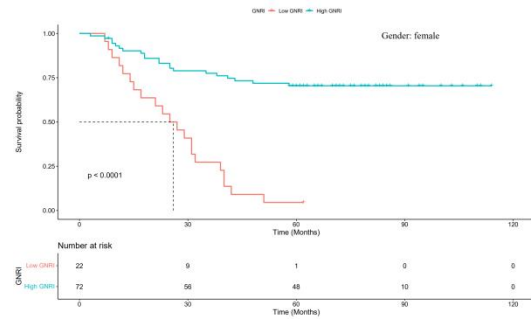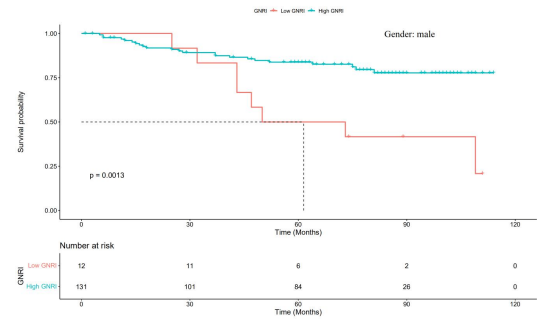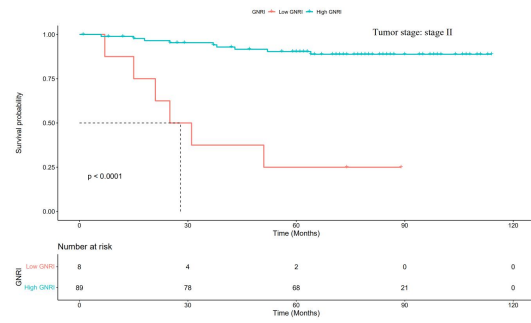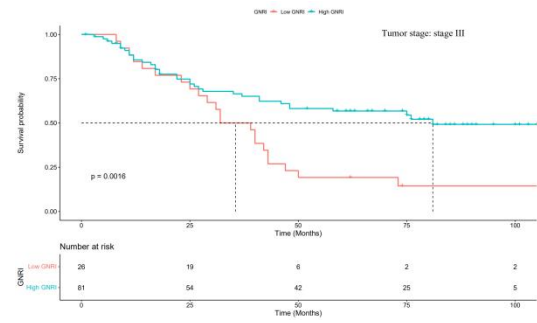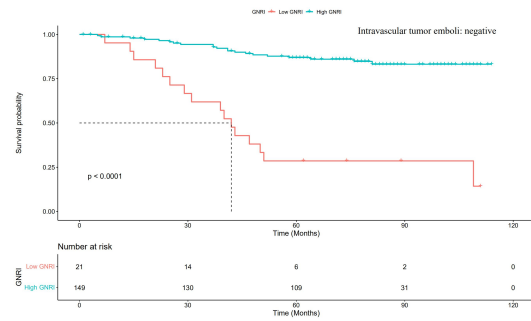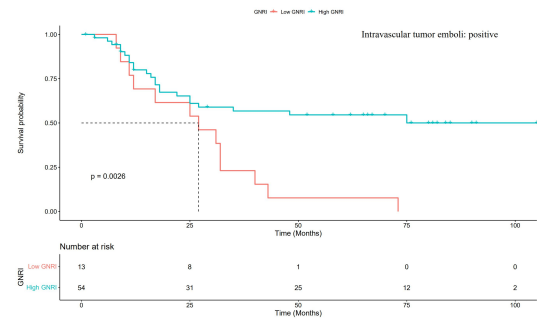

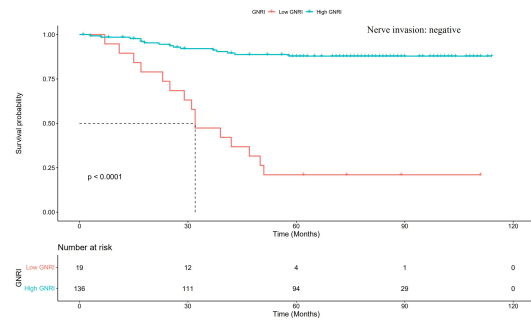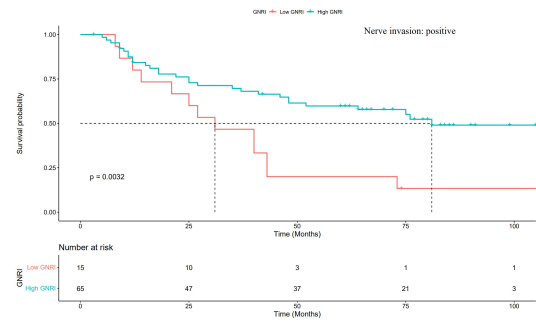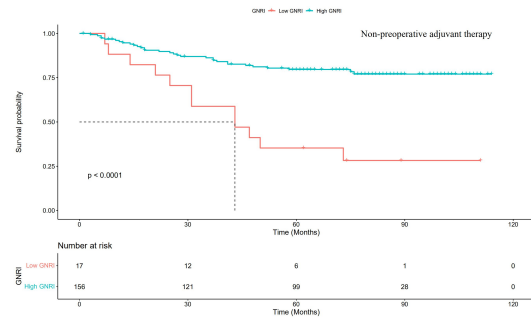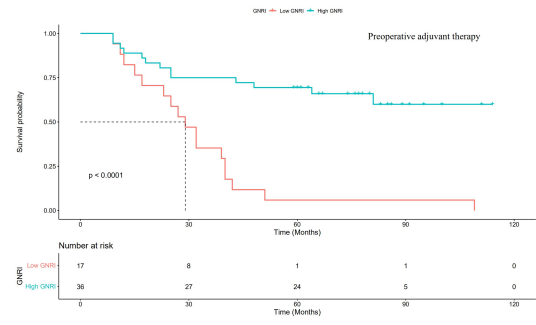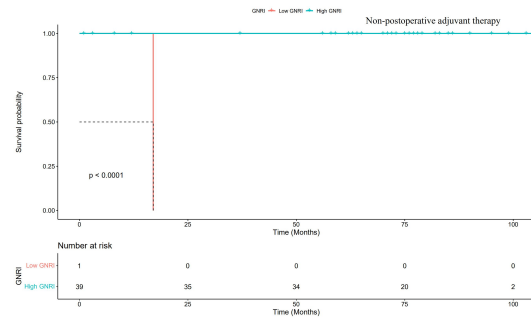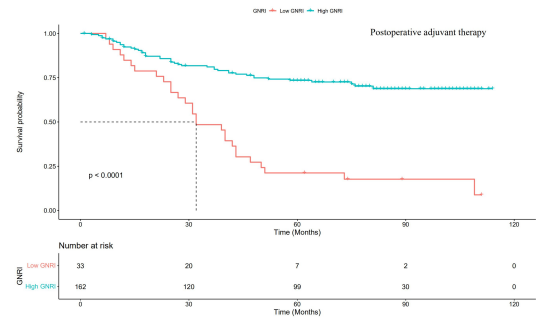

Supplement: SUPPLEMENTARY FIGURE S6 — Survival prediction of GNRI in different subgroups. [file Image_6.pdf]
