# Supplementary material for: Prognostic value of systemic immune inflammation index and geriatric nutrition risk index in early-onset colorectal cancer
Source: Front Nutr. 2023 Apr 18;10:1134300. doi: 10.3389/fnut.2023.1134300 (PMC10151795; doi:10.3389/fnut.2023.1134300)
Supplement: Supplementary file 7 [file Table_1.docx]

| **Variables** | 1. **Index** | **95% CI** |
| --- | --- | --- |
| NLR | 0.666 | 0.601-0.731 |
| PLR | 0.624 | 0.560-0.688 |
| ALI | 0.626 | 0.563-0.688 |
| SII | 0.692 | 0.633-0.750 |
| GNRI | 0.711 | 0.652-0.770 |
| AGR | 0.586 | 0.517-0.655 |
| PNI | 0.638 | 0.564-0.712 |

**TableS1 The c-index of different inflammatory indexes.**

Notes: NLR: neutrophil to lymphocyte ratio; PLR: platelet to lymphocyte ratio; ALI: advanced lung cancer inflammation index; SII: systemic immune inflammation index; GNRI: geriatric nutrition risk index; AGR: albumin to globulin ratio; PNI: prognostic nutritional index; CI, confidence interval.
