# Supplementary material for: Prognostic value of systemic immune inflammation index and geriatric nutrition risk index in early-onset colorectal cancer
Source: Front Nutr. 2023 Apr 18;10:1134300. doi: 10.3389/fnut.2023.1134300 (PMC10151795; doi:10.3389/fnut.2023.1134300)
Supplement: Supplementary file 8 [file Table_2.docx]

**Table 2 Univariate and multivariate analysis on the OS of SII.**

| **Variables** | **OS (model 0)^a^** |  | **OS (model 1)^b^** |  | **OS (model 2)^c^** |  |
| --- | --- | --- | --- | --- | --- | --- |
|  | **Crude HR (95%CI)** | **Crude P** | **Adjusted HR (95%CI)** | **Adjusted P** | **Adjusted HR (95%CI)** | **Adjusted P** |
| As continuous (per SD) | 1.07 (1.04-1.09) | 0.000^*^ | 1.05 (1.03-1.10) | 0.000* | 1.08 (1.05-1.11) | 0.000* |
| By SII cut-off  ≤637.6  ＞637.6 | /  5.27 (2.91-9.54) | /  0.000* | /  4.22 (2.29-7.77) | /  0.000* | /  4.42 (2.36-8.27) | /  0.000* |
| By SII interquartile  Q1 (~437.93)  Q2 (437.93-691.19)  Q3 (691.19-890.71)  Q4 (890.71~) | /  3.74 (1.38-10.16)  5.46 (2.08-14.33)  9.43 (3.69-24.09) | /  0.009*  0.001*  0.000* | /  3.57 (1.31-9.75)  3.93 (1.47-10.48)  7.19 (2.79-18.55) | /  0.012*  0.006*  0.000* | /  4.09 (1.47-11.37)  3.97 (1.45-10.86)  8.49 (3.22-22.36) | /  0.006*  0.007*  0.000* |

Notes: SII, systemic immune inflammation index ; OS, overall survival; HR, hazards ratio; CI, confidence interval; a Model 0: Unadjusted. b Model 1: Adjusted for age, gender, BMI and tumor stage. c Model 2: Adjusted for age, gender, BMI, tumor stage, smoking, alcohol, tumor location, differentiated degree, nerve invasion, intravascular tumor emboli, preoperative therapy and postoperative therapy.
