# Supplementary material for: Prognostic value of systemic immune inflammation index and geriatric nutrition risk index in early-onset colorectal cancer
Source: Front Nutr. 2023 Apr 18;10:1134300. doi: 10.3389/fnut.2023.1134300 (PMC10151795; doi:10.3389/fnut.2023.1134300)
Supplement: Supplementary file 9 [file Table_3.docx]

| **Variables** | **OS (model 0)** |  | **OS (model 1)** |  | **OS (model 2)** |  |
| --- | --- | --- | --- | --- | --- | --- |
|  | **Crude HR (95%CI)** | **Crude P** | **Adjusted HR (95%CI)** | **Adjusted P** | **Adjusted HR (95%CI)** | **Adjusted P** |
| As continuous (per SD) | 0.96 (0.95-0.97) | 0.000* | 0.97 (0.96-0.98) | 0.000* | 0.97 (0.96-0.98) | 0.000* |
| By GNRI cut-off  ≤83.1  ＞83.1 | /  0.23 (0.14-0.36) | /  0.000* | /  0.35 (0.21-0.58) | /  0.000* | /  0.33 (0.19-0.56) | /  0.000* |
| By GNRI interquartile  Q1 (~91.50)  Q2 (91.50-100.37)  Q3 (100.37-107.64)  Q4 (107.64~) | /  0.67 (0.41-1.13)  0.23 (0.12-0.46)  0.21 (0.10-0.44) | /  0.134*  0.000*  0.000* | /  0.85 (0.50-1.47)  0.30 (0.15-0.61)  0.28 (0.13-0.62) | /  0.582  0.001*  0.002* | /  0.70 (0.39-1.23)  0.29 (0.14-0.59)  0.29 (0.13-0.64) | /  0.219  0.001*  0.002* |

**Table3 Univariate and multivariate analysis on the OS of GNRI**

Notes: SII, GNRI, geriatric nutrition risk index; OS, overall survival; HR, hazards ratio; CI, confidence interval; a Model 0: Unadjusted. b Model 1: Adjusted for age, gender, BMI and tumor stage. c Model 2: Adjusted for age, gender, BMI, tumor stage, smoking, alcohol, tumor location, differentiated degree, nerve invasion, intravascular tumor emboli, preoperative therapy and postoperative therapy.
